# Reactive oxygen species‐induced SIAH1 promotes granulosa cells' senescence in premature ovarian failure

**DOI:** 10.1111/jcmm.17264

**Published:** 2022-03-09

**Authors:** Li Lin, Wujiang Gao, Yumei Chen, Taoqiong Li, Chunli Sha, Lu Chen, Meiling Yang, Hong Wei, Yunpeng Chen, Xiaolan Zhu

**Affiliations:** ^1^ Reproductive Center The Fourth Affiliated Hospital of Jiangsu University Zhenjiang China; ^2^ Department of Central Laboratory The Fourth Affiliated Hospital of Jiangsu University Zhenjiang China; ^3^ Department of Radiology The Affiliated Hospital of Jiangsu University Zhenjiang China; ^4^ International Genome Center of Jiangsu University Zhenjiang China

**Keywords:** premature ovarian failure, reactive oxygen species, senescence, SIAH1, telomere

## Abstract

Reactive oxygen species (ROS) exposure triggers granulosa cells' (GCs) senescence, which is an important causal factor for premature ovarian failure (POF). However, underlying mechanism in this process remains unknown. In our study, we observed increased ROS levels in POF ovarian tissues, POF patient follicular GCs and cyclophosphamide (CTX) pretreated GCs. Correspondingly, increased SIAH1, reduced TRF2 and GC senescence were also found in these cases. Silencing of SIAH1 rescued ROS‐induced TRF2 reduction and cell senescence in GCs. Moreover, SIAH1 co‐localized with TRF2 in the cytoplasm, facilitating its ubiquitination degradation, further leading to telomere abnormalities in GCs. In conclusion, our findings indicate that ROS induces telomere abnormalities by augmenting SIAH1‐mediated TRF2 degradation, leading to cell senescence in GCs in POF processing.

## 1 INTRODUCTION

Premature ovarian failure (POF) is a common disease in women, characterized as abnormal declines in ovarian follicle numbers and oocyte quality. Patients have early depletion of primordial follicle pool associated with amenorrhea before the age of 40, which could be diagnosed by elevated serum FSH levels (>40 IU/L).^1^, ^2^ Causes of this disease could be mitochondrial dysfunction, auto‐immune, chemotherapeutic treatments and cell senescence.^3^ During ageing and senescence, accumulation of endogenous reactive oxygen species (ROS) and weakened antioxidant defences promoted oxidative damage in cell structures, rendering membranal lipid peroxidation, enzyme inactivation, protein oxidation and DNA damage.^4^, ^5^ As stated in the widely acknowledged ‘free radical theory’, oxidative damage continuously occurs during ageing and ROS is the driving force of ageing and cell senescence, whereas ageing is also a result of oxidative damage.^6^, ^7^ In addition, crosstalk between oocytes and GCs regulates oocytes competency and female fertility, deciding prognosis of assisted reproduction treatment (ART).^8^ Therefore, to elucidate the underlying mechanism of oxidative cell senescence‐driven pathological process in GCs beside oocytes is necessary for comprehensively understanding POF pathogenesis.

Ubiquitination plays a central role in selective degrading damaged proteins in various forms.^9^ The ubiquitin–proteasome pathway is also an important defence machinery against oxidative damage.^10^ SIAH1, one of the seven members in absentia homologue family, is a critical E3 ubiquitin ligase which prominently controls a variety of biological processes, including transcription, mitosis and cell migration.^11^, ^12^ SIAH1 also participates in ubiquitination degradation of proteins involved in tumorigenesis (Bcl‐2, JNK and EHMT2),^13^, ^14^, ^15^ DNA repair (HIPK2, beta‐catenin)^16^, ^17^, ^18^ and hypoxia response (PHD3, FIH).^19^, ^20^ It is reported that SIAH1 expression could be promoted by E2F1, which commonly induced by ROS.^21^ However, it is remained elusive whether SIAH1 is a target of ROS in GC senescence, and whether this process promotes GC senescence in POF initiation.

Telomeres are composed of TTAGGG repeats as the natural ends of chromosomes.^22^, ^23^ Its dysfunction is the origin of replicative cell senescence in ageing.^24^ TRF2 is a telomere‐binding protein, playing a vital role in t‐loop generation, protecting chromosomes ends from being mis‐recognized as double‐strand DNA breaks by DNA damage repair system.^25^ Early studies found TRF2 was associated with the normality of double‐stranded telomeric DNA structure, telomere morphology and function.^26^ TRF2 knockdown led to t‐loop breakdown and initiated a rapid dsDNA break response, p53‐p21 driven cell cycle arrest and cell senescence.^27^ Fu et al. reported a proteolytic regulation of TRF2 by SIAH1 in human fibroblasts.^28^ These facts support that SIAH1 may be involved in ROS‐mediated GC senescence, and this regulatory path may be developed as novel therapeutic targets for rescuing ovarian function in POF patients.

## 2 MATERIALS AND METHODS

### 2.1 Patients and GC isolation, and serum protein extraction

This study was approved by the Ethics Committee of the Fourth Affiliated Hospital of Jiangsu University. Informed consents were obtained from all POF patient participants at the Reproductive Medical Center of the Fourth Affiliated Hospital of Jiangsu University, including patients received in vitro fertilization or intracytoplasmic sperm injection‐ embryo transfer (IVF/ICSI‐ET). Ovarian GCs were isolated from follicular fluid, centrifuged at 280 *g* for 10 min and re‐suspended in phosphate‐buffered saline containing 0.1% hyaluronidase (#H1136‐1AMP; Sigma) at 37°C for 30 min. Serum protein was extracted from patient serum with RAPI lysis and sulfhydryl reducing agent. Supernatant was collected after 12,000 *g* centrifugation for 15 min, denatured and then frozen for further examination.

### 2.2 Cell culture and transfection

Human granulosa‐like tumour cell line KGN was obtained from Shanghai Ji he Biotechnology Co, LTD. Cells were cultured with Dulbecco's modified Eagle's medium (DMEM)/HamF‐12 growth medium (#11320033; GIBCO BRL) +10% fetal bovine serum (#10099141C；GIBCO BRL) +1% penicillin‐streptomycin (#15240062; GIBCO BRL). Cells were authenticated by Short Tandem Repeat assay (STR). For temporal overexpression, 2 µg plasmids were transfected into KGN cells and GCs seeded into six‐well plates (5 × 10^5^ cells/well) with 5 µl Lipo2000 (#11668019; Invitrogen) according to manufacturer's protocol. Transfection efficiency was assessed by Western blot at 48 h post‐transfection. All plasmid information was described in Supplementary Material 1.2.

### 2.3 In vitro ubiquitination and co‐immunoprecipitation

293T cells were transfected with plasmids. 36 h later, cells were pretreated with 15 μM MG132 (#HY‐13259; MedChemExpress) for 6 h and then lysed with IP lysis buffer. Whole‐cell lysates were incubated with indicated antibody overnight at 4°C and then immunoprecipitated with Protein A/G beads (#CS203178; Millipore). Immunoprecipitates were washed five times, and then, protein was eluted for Western blotting analysis.

### 2.4 Immunohistochemical

Isolated ovarian tissues were fixed with 4% paraformaldehyde, embedded in paraffin and then sliced in 4 μm thickness. After dehydration with gradient alcohol and xylene, sections were incubated with anti‐SIAH1(#ab2237; Abcam) antibody at 4℃ in humid and then incubated with the secondary antibody at room temperature for 2 h.

### 2.5 Immunofluorescence

Cells were rinsed with PBS and fixed with 4% PFA at room temperature for 20 min. Then, cells were permeabilized with 0.1% Triton X‐100 and blocked with 1% bovine serum albumin in TBS for 1 h. Next, cells were incubated with specific primary antibody at 4℃ overnight and with secondary antibodies for 1 h at room temperature in dark. Nucleus was stained with DAPI (Beyotime) for 10 min at room temperature before imaging.

### 2.6 Electron microscopy

At Day 21 post‐BMSC transplantation, two rats were randomly taken from negative control, model control and BMSC transplantation group. Then, rats were sacrificed for dissection. Indicated tissue was fixed with 2.5% glutaraldehyde phosphate buffer +1% osmic acid fixative. After dehydration and embedding, tissue blocks were subjected to ultra‐thin sectioning. Sections were stained with lead citrate and washed with ultra‐pure water for image capture and analysis by transmission electron microscopy.

### 2.7 Measurement of ROS

Redox‐sensitive, cell‐permeable fluorophore DHE (#KGAF019; KeyGen biotech) was used to measure superoxide production in situ. DHE (10 mM) was incubated with unfixed frozen sections or cultured cells in a light‐protected humidified chamber at 37°C for 30–60 min. Incubated samples were washed with PBS for three times, mounted with fluorescent mounting medium (DAKO) and then stained with 10 µM DCFH‐DA (#D6470; Solarbio). Images in similar exposure conditions were picked for fluorescence semi‐quantification. The grayscale was detected as the signal intensity, and the average in group was compared. For each group, 10 randomly picked sights were included for statistical analysis.

### 2.8 Senescence‐associated β‐galactosidase activity

Cell senescence in KGN and GCs were measured by staining with Senescence β‐Galactosidase Staining Kit (#C0602, Beyotime). Briefly, cells were washed with PBS and fixed with β‐galactosidase fixing solution for 15 min at room temperature. After another three times washing, cells were water bathed in 37°C with freshly prepared senescence‐associated β‐galactosidase activity (SA‐β‐gal) staining solution overnight. In each view, at least 300 cells were measured.

### 2.9 Metaphase chromosome spreads and telomere‐PNA FISH analysis

After 0.1 μg/ml colchicine (Beyotime, SC7992‐10 mM) incubation at 37°C for 3 h, cell cycle was arrested in metaphase as expected. All cells were collected by gently pipetting, washed once with 1 × PBS and incubated with 0.075 M KCl at 37°C for 30 min. After incubation, cells were fixed with 3:1 methanol/glacial acid and 0.075 M KCl (1:4 fixative/0.075 M KCl) for 10 min. The fixation process was repeated for three times. Then, cells were dropped onto a wet glass slide and dried in a 75°C oven. After 5 min rehydration with PBS, all slides were fixed with chromosome diffusion +2% paraformaldehyde for 10 min. Next, slides were treated with pepsin (#P8390; Solarbio) at 37°C and then washed with PBS twice, 5 min each round. Then, the slides were sequentially treated with 70%, 85% and 100% ethanol, 2 min each step, and dried for at least 30 min. Next, 200 μl hybridization buffer (2% BSA 10 μl, 0.8 μl final concentration 125 nmol Telc‐FITC (#200616PL‐01; panagene), 0.6 × SSC 20 μl, deionized formamide 140 μl, add ddH_2_O 56.4 µl) was added on slides. Before staining, slides were heated at 85°C for 3 min for DNA denaturing and then incubated at 37°C for 2 h. After washing with washing buffer I (10 mM Tris, 70% formamide, 2 × 15 min) and 0.1% PBST (3 × 5 min), nucleus was stained with DAPI, and slides were mounted with glycerol and imaged by fluorescent confocal microscopy.

### 2.10 Statistical analysis

All experiments have repeated at least three times. Statistical analysis was performed with SPSS 13.0 (IBM) and GraphPad Prism 6 (GraphPad Software). Unless otherwise noted, difference was analysed by the two‐tailed Student's *t*‐test or one‐way ANOVA test with Tukey's post hoc analysis (for comparing more than two groups). Differences were defined as significant when *p* < 0.05. Data were shown as mean ± SD.

## 3 RESULTS

### 3.1 ROS accumulation accelerates GC senescence during ovarian ageing

To elucidate the connection between ROS accumulation and oocyte quality decline in ovarian ageing, we first measured ROS in POF in vitro and in vivo. POF rat model was established as previously described.^29^ As shown, we observed dysregulated oestrus cycle, increased FSH and LH levels, and decreased AMH values, corresponding with more active apoptosis and decreased ovarian reserve (Figure S1). In addition, pathologic phenotypes in GCs including dissolved nucleus, nuclear membranes loss, pyknotic chromatin, edge aggregation, apoptotic bodies and vacuolated mitochondria were all observed. We also detected excessive ROS accumulation in POF rat ovaries, along with downregulation of antioxidant genes (such as SOD2, CAT and GLUT1) (Figure S3). We previously reported potential therapeutic function of BMSCs in POF. Here, we validated ovarian function restoration and restricted ROS deposition after BMSC transplantation. Consistently in POF cell model, ROS also accumulated in CTX‐treated GCs. These in vitro cultured GCs were validated by FSHR immunofluorescence. ROS level and SA‐β‐gal+ cells were significantly increased upon CTX treatment, implying a strong cell senescent status. Oppositely, BMSC coculture greatly reduced ROS accumulation and SA‐β‐gal+ cell proportion (*p* < 0.05).

To figure out the role of ROS played in cell senescence in this case, we pretreated GCs with H_2_O_2_ for 2 h. The ratio of SA‐β‐gal positive cells in total GCs was significantly increased, confirming that ROS could effectively induce GC senescence. ROS scavenger N‐acetyl‐L‐cysteine (NAC) restrained ROS accumulation in GCs upon H_2_O_2_ treatment. Consistently, the occurrence of GC senescence was significantly reduced by NAC. These results supported that GCs underwent excessive oxidative stress and cell senescence in POF in vivo and in vitro, and ROS is a major factor contributing to this process.

### 3.2 ROS promotes GC senescence via upregulating SIAH1

**FIGURE 1:**
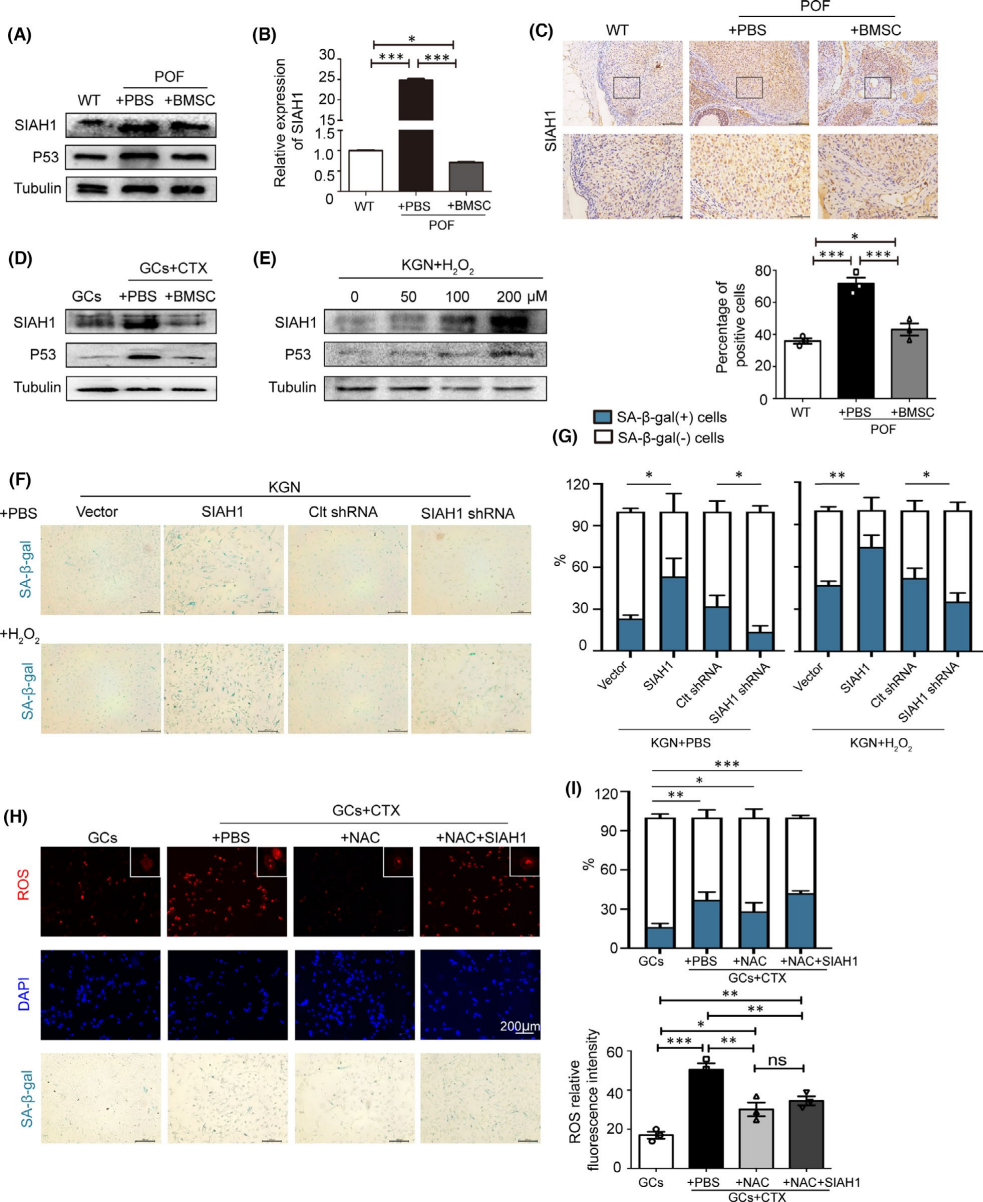
ROS promotes GC senescence via upregulating SIAH1. (A) Protein levels of SIAH1 and P53 in POF ovarian tissues were assessed by immunoblots. (B) SIAH1 mRNA level was determined by RT‐qPCR with normalization to Actin. The data are presented as medians ± interquartile range. ***p < 0.001. (C) Immunohistochemistry of SIAH1 in ovarian tissues. Positive cells were counted from 10 randomly picked sights. (D) SIAH1 and P53 protein levels in CTX‐treated GCs with or without coculturing with BMSCs. (E) SIAH1 and P53 protein levels in indicated H_2_O_2_‐treated KGN cells. (F) Cell senescence was detected by SA‐β‐gal staining in KGN cells with transfection of empty plasmids or expressing SIAH1, Clt shRNA or SIAH1 shRNA upon 100 μM H_2_O_2_ treatment. (G) Quantification of SA‐β‐gal positive cells in (F). Error bars indicate SD, *n* = 3, ***p < 0.001, **p < 0.01, *p < 0.05. (H) CTX‐GCs were treated as indicated, and (I) SA‐β‐gal positive cells and ROS level were quantified, Error bars indicate SD, *n* = 3, ***p < 0.001, **p < 0.01, *p < 0.05. CTX, cyclophosphamide; GCs, granulosa cells; POF, premature ovarian failure; ROS, Reactive oxygen species

In order to clarify whether SIAH1 was involved in POF development, we measured SIAH1 expression level in POF in vivo and in vitro. As shown in Figure 1A,B, SIAH1 mRNA and protein levels were elevated in POF rat ovaries and CTX‐treated GCs, whereas back to normal upon BMSC treatment (Figure 1C,D). Upon H_2_O_2_ inducing ROS in the GC line KGN, SIAH1 and P53 protein level considerably increased, accompanied with more senescent cells (Figure 1E). In contrast, silencing of SIAH1 with shRNA effectively protected cells from ROS‐induced senescence, suggesting SIAH1 may be a key mediator in regulating this process (Figure 1F). In CTX‐treated GCs, again we confirmed that SA‐β‐gal signal was significantly reduced by NAC, but notably, upregulating SIAH1 re‐induced SA‐β‐gal signal in NAC‐treated cells (Figure 1H), implying a vital role SIAH1 played in GC senescence.

### 3.3 TRF2‐mediated SIAH1 function on regulating ROS‐induced GC senescence

**FIGURE 2:**
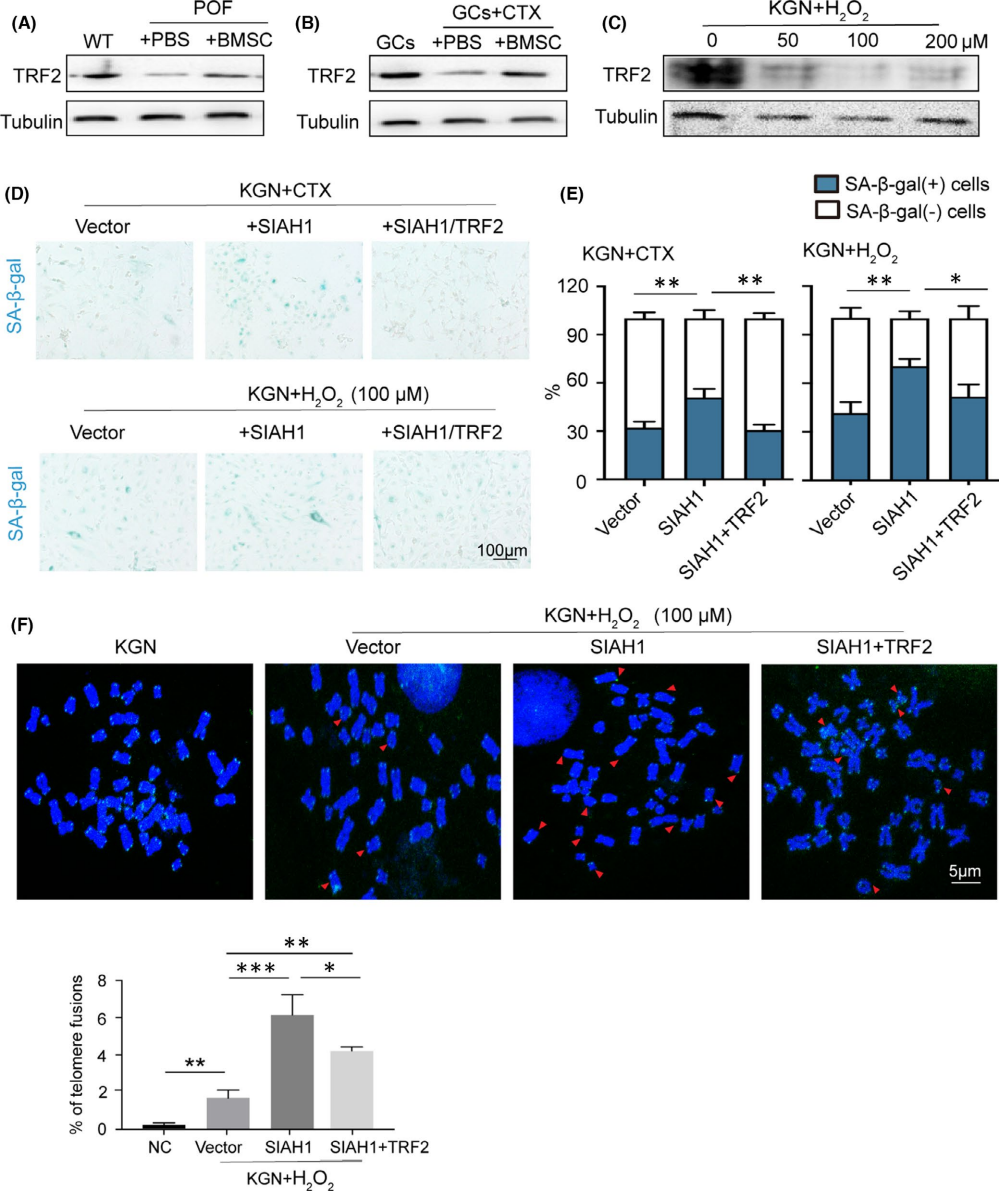
TRF2‐mediated SIAH1 function on regulating ROS‐induced GC senescence. (A) TRF2 protein levels in POF ovarian tissues were determined by immunoblots. (B) TRF2 and SIAH1 protein levels were detected in CTX‐treated GCs with or without coculturing with BMSCs. (C) TRF2 protein levels in KGN cells with indicated H_2_O_2_ treatment. (D,E) SA‐β‐gal staining and quantification in KGN with indicated treatment. (F) Metaphase chromosome in KGN cells of empty plasmids or expressing SIAH1 or SIAH1+TRF2. Telomeric DNA was stained in green among DAPI (blue) stained DNA. Red arrows indicate telomere with fusions or malformation. Bar graph shows the ratio of telomere fusion in 2000 chromosomes. Error bars indicate the SD, *n* = 3, ***p < 0.001, **p < 0.01, *p < 0.05. CTX, cyclophosphamide; GCs, granulosa cells; POF, premature ovarian failure; ROS, Reactive oxygen species

SIAH1 was previously reported promoting TRF2 degradation in human fibroblasts,^28^ while TRF2 has a crucial function on telomere protection.^30^ Therefore, we extended our study to assessed whether TRF2 and telomere dysfunction were involved in SIAH1‐regulated GC senescence upon ROS treatment. As showed, TRF2 protein level was decreased in both of POF rats and CTX‐GCs model (Figure 2A,B). Furthermore, SIAH1 expression elevated by increasing H_2_O_2_ concentration, whereas TRF2 protein stability decreased (Figure 2C). As shown in Figure 2D, overexpression of SIAH1 dramatically promoted cell senescence while co‐expression with TRF2 had the opposite function. Moreover, in H_2_O_2_‐pretreated cells, TRF2 also restrained SIAH1‐induced senescence (Figure 2E). By telomeric PNA‐FISH with GCs in metaphase, we analysed the impact of SIAH1/TRF2 to telomeric DNA. As showed, SIAH1 significantly induced telomere fusions and other chromosomal abnormalities in GCs (Figure 2F), which could be reversed by TRF2 overexpression. In summary, TRF2‐mediated telomere protection might be involved in the process of SIAH1 inducing GC senescence.

### 3.4 SIAH1 mediates TRF2 protein ubiquitination degradation

**FIGURE 3:**
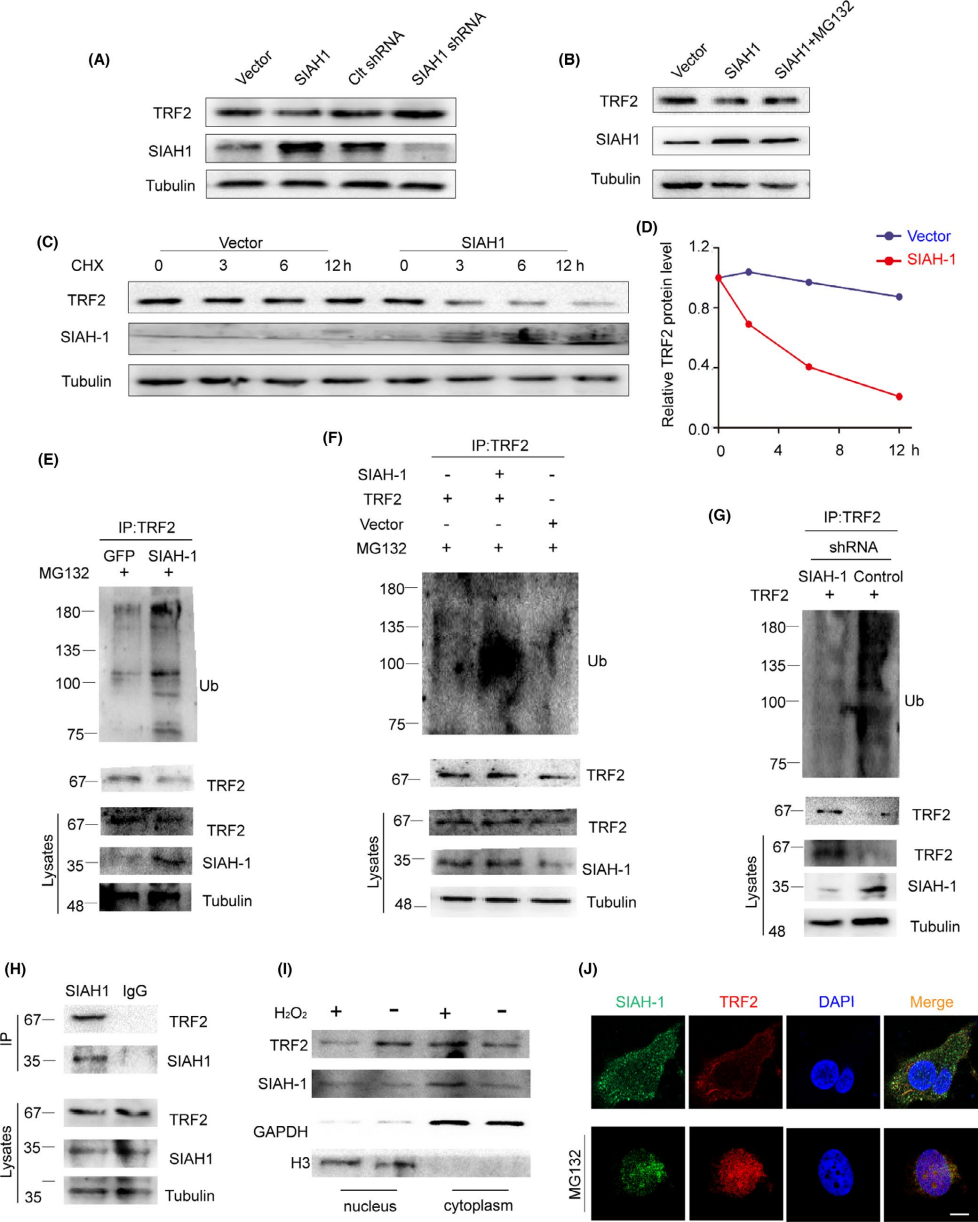
E3 ubiquitin ligase SIAH1 promotes TRF2 ubiquitination degradation in vivo. (A) KGN cells were transfected with indicated plasmids. SIAH1 and TRF2 protein levels were detected by immunoblots. (B) KGN cells were transfected with empty plasmids or plasmids expressing SIAH1. 36h later, cells were pretreated with or without MG132 for 6 h before examining by immunoblots. (C) KGN cells with or without SIAH1 overexpression were treated with cycloheximide and indicated proteins were checked by immunoblots. (D) Measurements of TRF2 protein bands in C. (E) Ubiquitination and total protein level of endogenous TRF2 were checked in cells with or without SIAH1 overexpression. (F) HEK293T cells were transfected with indicated plasmids. 36 h later, cells were treated with MG132 for 6 h before immunoprecipitation. Indicated proteins were examined by immunoblots. (G) HEK293T cells were transfected with control or SIAH1 shRNA. Protein lysates were detected by Western blot. (H) Co‐immunoprecipitation of TRF2 protein by SIAH1 and IgG. Indicated protein levels were checked by immunoblots. (I) Immunoblots of SIAH1 and TRF2 in nucleus and cytoplasm fractions in cells. (J) Plasmids expressing GFP‐SIAH1 or FLAG‐TRF2 were transfected to KGN cells. Their localization was examined by immunofluorescence. Cell nuclei were marked by DAPI. CTX, cyclophosphamide; GCs, granulosa cells; POF, premature ovarian failure; ROS, Reactive oxygen species

Inspired by opposite trends of SIAH1 and TRF2 expression in POF, we set out to investigate the underlying mechanism of SIAH1 regulating TRF2. SIAH1 participated in ubiquitin‐mediated proteolytic cascade, so we tested whether SIAH1 serves as an E3 ubiquitin ligase in TRF2 ubiquitination. It was found that TRF2 protein level was significantly reduced by SIAH1 (Figure 3A), but this effect could be attenuated by MG132 (a proteasome inhibitor) (Figure 3B), suggesting that TRF2 may be SIAH1's substrate. To further identify whether SIAH1 is the responsible E3 ligase of TRF2, KGN overexpressed with SIAH1 were treated with combination of cycloheximide (CHX, an inhibitor of protein biosynthesis). Measurement of immunoblots confirmed that SIAH1 significantly reduced TRF2 protein stability (Figure 3C,D). With suppressing TRF2 degradation, we observed SIAH1 significantly enhanced TRF2 ubiquitination (Figure 3E,F). In contrast, silencing of SIAH1 inhibited TRF2 ubiquitination (Figure 3G). All above results indicate that SIAH1 destabilizes TRF2 proteins in a ubiquitin–proteasome pathway.

Next, we verified protein–protein interaction of endogenous SIAH1 and TRF2 by CO‐IP (Figure 3H). Interestingly, nuclear localization of TRF2 was reduced upon H_2_O_2_ while cytoplasmic TRF2 increased (Figure 3I), which was consistent with the fact that ubiquitinated TRF2 protein would be exported to cytoplasm.^28^ In further validation, we showed TRF2 was retained in nucleus upon MG132 treatment by measuring colocalization signals. With withdrawing MG132, TRF2 was exported to cytoplasm and co‐localized with SIAH1, and then underwent degradation (Figure 3J, Figure S4C). These results indicate that SIAH1 promotes TRF2 ubiquitination degradation in the cytoplasm upon ROS.

### 3.5 SIAH1 expression elevated in POF patient GCs

**FIGURE 4:**
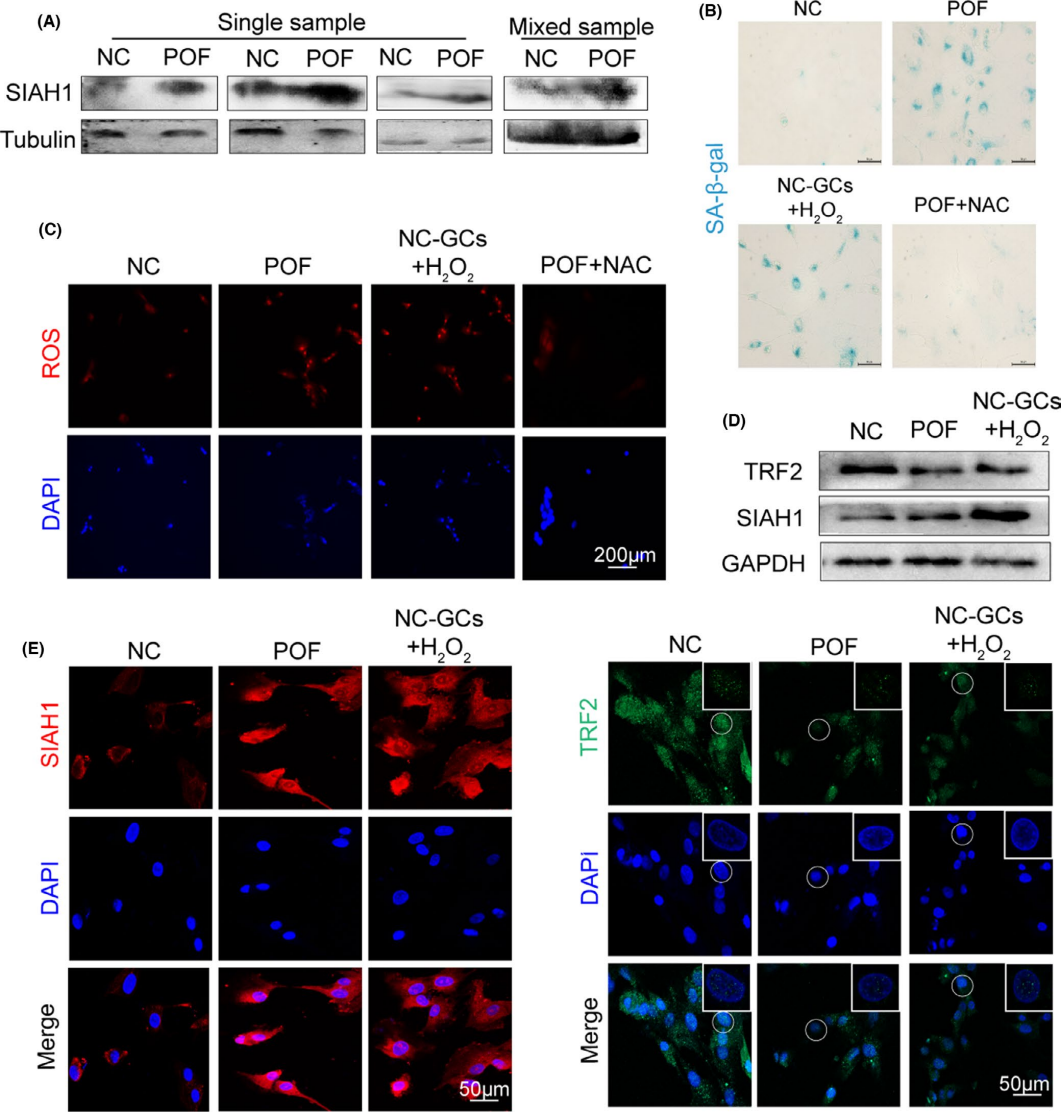
Validating ROS‐p53‐SIAH1‐TRF2 signalling axis in POF patient granulosa cells. (A) Immunoblots of SIAH1 in serum GCs in POF patients or healthy controls. (B) SA‐β‐gal staining in GCs isolated from POF patients and controls, or in cultured GCs with 100 μM H_2_O_2_ treatment. (C) ROS (red) was detected by DHE in GCs from POF patients or H_2_O_2_‐treated GCs. (D) Immunoblots of SIAH1 and TRF2 proteins in NC, POF and H_2_O_2_‐treated patients' GCs. (E) Immunofluorescence of SIAH1 and TRF2 foci in GCs from the NC, POF individuals or H_2_O_2_‐treated GCs. CTX, cyclophosphamide; GCs, granulosa cells; POF, premature ovarian failure; ROS, Reactive oxygen species

To check clinical relevance of the ROS‐p53‐SIAH1‐TRF2 axis in POF, we assessed ROS levels, cell senescence and SIAH1 expression level in both serum and GCs isolated from 10 idiopathic POF patients and 10 age‐matched healthy females. These individuals were undergoing in vitro fertilization and embryo transfer (IVF‐ET) treatment. Increased SIAH1 expression was found in POF patient serum (Figure 4A) and cumulus GCs. POF GCs also had lower TRF2 level, a senescence‐associated phenotype and high ROS accumulation (Figure 4B–E). Comparing to healthy controls, H_2_O_2_ reduced TRF2 and promoted cell senescence in POF patient derived GCs (Figure 4B–E). Collectively, POF patient GCs had obvious senescence phenotype along with high ROS accumulation and SIAH1 expression level, implying that SIAH1 could be a potential therapeutic target for POF.

## 4 DISCUSSION

Our study reported that elevated ROS promoted SIAH1 expression in POF in both in vivo and in vitro cases. We also confirmed this process in clinical human samples. As known, SIAH1 was well‐studied in hypoxia, ageing and DNA damage with identification of numerous SIAH1‐interacting proteins. Our results indicated that SIAH1 responded to ROS and promoted GC senescence in POF. Our data also demonstrated that SIAH1 directly bound with TRF2 to promote its ubiquitination and degradation, rendering telomere abnormalities and cell senescence in GCs, subsequently contributing to POF development.

Clinically, POF patients have significant early exhaustion of ovarian follicles along with amenorrhea, hypoestrogenism and elevated gonadotropin levels before age 40.^2^ Due to premature atresia of follicles and the arrest of spontaneous ovulation, the chances of conceiving are very low in POF patients.^31^ However, POF pathogenesis has not been fully interpreted so far. Numerous studies demonstrated that reproductive capacity was very sensitive to redox homeostasis and oxidant enzyme levels.^32^ A widely acknowledged theory of follicle senescence emphasized that ROS induced GCs degeneration as a critical causative factor,^33^ resulted in reduced follicle numbers, compromised follicle quality and aberrant reproductive endocrinology in ovaries.

Reactive oxygen species is a double‐edged sword in oocyte development. It is fundamental in mediating folliculogenesis, meiosis, ovulation and embryonic development as secondary messengers in signalling transduction,^34^ whereas excessive ROS will attacks DNA, proteins and lipids within the mitochondria, inducing granulosa cell apoptosis, chromosomal non‐disjunction, oocyte ageing and a series of reproductive diseases.^35^, ^36^, ^37^ Here, we identified an E3 ubiquitin ligase as a new and effective target which was expected to reach the goal to reverse the ROS‐induced fertility decline in POF patients. We identified p53 induced SIAH1 as an important regulatory factor to promote GCs senescence in POF. Consistently, SIAH1 induced senescence in KGN cells, and SIAH1 overexpression made cells more prone to H_2_O_2_‐induced senescence.

Reactive oxygen species vitiates the maturation and quality of oocytes through destroying the spindles in chromosome assembly, interrupting mitochondrial function, affecting motility during early meiosis by shortening telomeres.^38^, ^39^ TRF2 is responsible for the formation and maintenance of the ‘t‐loop’ structure,^25^ and TRF2 loss is associated with senescence‐related signalling.^40^ We found that when SIAH1 overexpression in GCs reduced the telomere protection factor TRF2 by promoting its ubiquitination. Telomeric DNA was continually consuming, resulting in chromosome‐end fusions and other types of damage. Consistently, in ROS‐driven GC senescence, ascending P53 and SIAH1 restrained TRF2 protein level and led to excessive chromosome end fusion and damage. This outcome was significantly avoided by exogenously expressed TRF2 (Figure 2G), confirming that TRF2 could protect telomere from fusions or other damage induced by SIAH1. It is reported that TRF2 decrease was associated with telomere dysfunction‐driven ageing acceleration. Supraphysiological amounts of TRF2 are also susceptible to chromosomal instability and impaired normal senescence checkpoint.^41^, ^42^ These non‐conflicting conclusions from different studies supported the fact that telomere regulation is an extremely elaborated, and both loss and excessive TRF2 lead to telomere dysfunction and chromosomal abnormalities.

## 5 CONCLUSION

**FIGURE 5:**
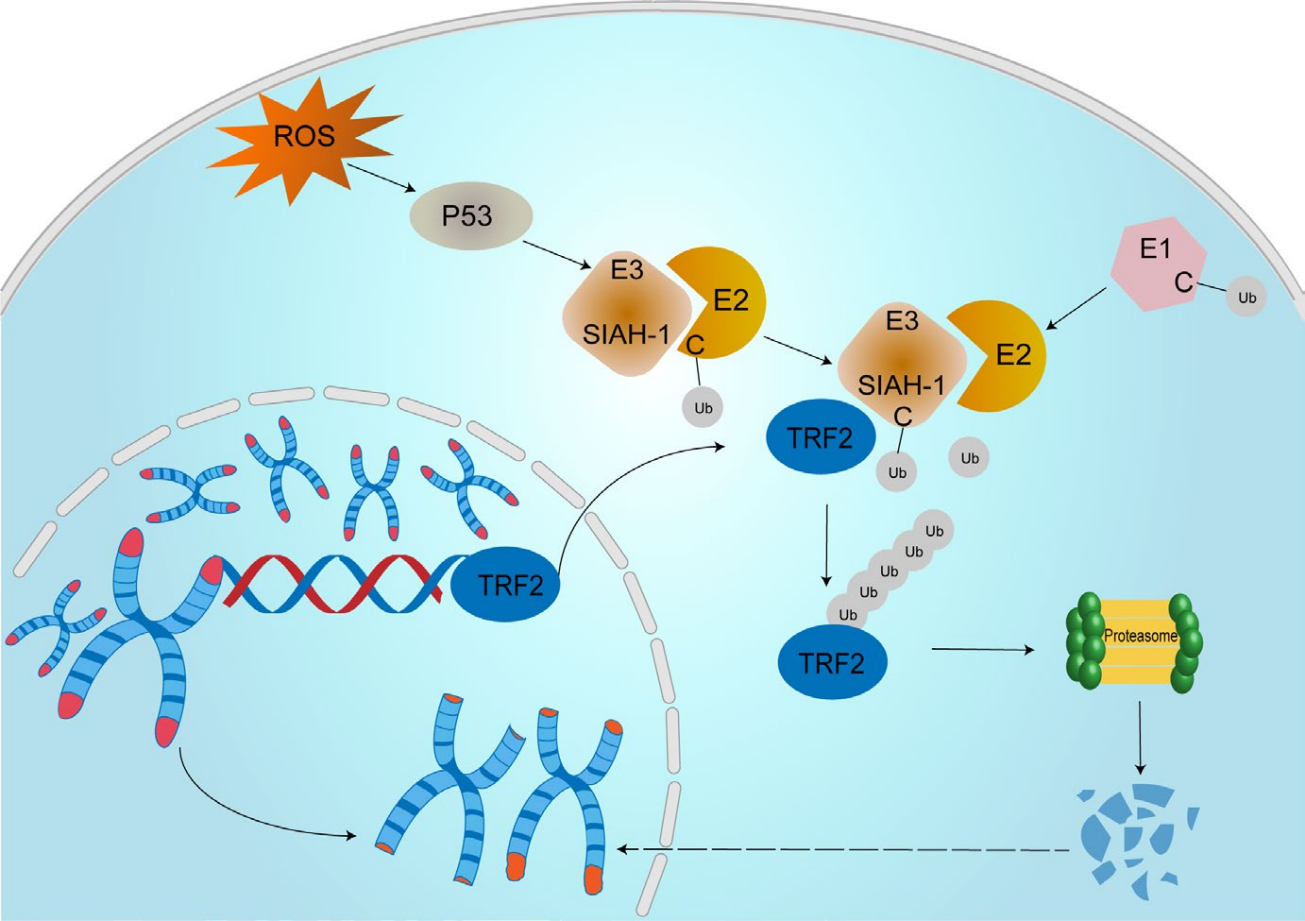
Overview of the ROS‐p53‐SIAH1‐TRF2 signalling axis in POF. The diagram shows the hypothetical model of ROS‐induced GC senescence in our study. Exogenous H_2_O_2_ induced intracellular ROS in GCs. ROS accumulation activated p53. Then, active P53 upregulated SIAH1 which promoted TRF2 ubiquitination degradation, resulting in GC senescence along with telomere shortening, loss, deforming. GCs, granulosa cells; POF, premature ovarian failure; ROS, Reactive oxygen species

In summary, our study revealed that SIAH1 mediated cytoplasmic TRF2 ubiquitination degradation and then induced telomere abnormalities, contributing to GC senescence upon oxidative stress. These findings implied SIAH1 might be developed into a novel therapeutical target to alleviate ROS‐induced GC senescence in POF (Figure 5). However, we also need additional investigation to in‐depth elucidate SIAH1 biological function for the goal of improving fecundity in POF in clinical.

## CONFLICT OF INTEREST

The authors declare no conflict of interest.

## AUTHOR CONTRIBUTIONS

**Li Lin:** Formal analysis (equal); Investigation (equal); Methodology (equal); Software (equal); Supervision (equal); Writing – original draft (equal). **Wujiang Gao:** Methodology (equal); Software (equal). **Yumei Chen:** Writing – original draft (equal). **Taoqiong Li:** Methodology (equal); Software (equal). **Chunli Sha:** Resources (equal); Supervision (equal). **Lu Chen:** Methodology (equal). **Meiling Yang:** Investigation (equal); Methodology (equal). **Hong Wei:** Supervision (equal); Visualization (equal); Writing – original draft (equal). **Yunpeng Chen:** Methodology (equal). **Xiaolan Zhu:** Conceptualization (lead); Funding acquisition (lead); Project administration (lead).

## Supporting information

Supplementary Material

## Data Availability

The raw data supporting the conclusions of this article will be made available by the authors, without undue reservation.
