# Supplementary material for: Reactive oxygen species‐induced SIAH1 promotes granulosa cells' senescence in premature ovarian failure
Source: J Cell Mol Med. 2022 Mar 9;26(8):2417–27. doi: 10.1111/jcmm.17264 (PMC8995443; doi:10.1111/jcmm.17264)
Supplement: Supplementary file 1 — Supplementary Material [file JCMM-26-2417-s001.docx]

Supplementary Material

# Supplementary Data

**1 Materials and methods**

**1.1 Experimental animals and BMSC transplantation**

Our experimental animals were female clean-grade Sprague Dawley (SD) rats weighing ~150g provided by the laboratory animal center of Jiangsu University. The rats were housed at room temperature (25 ± 2 °C) with a humidity of 45–55% and a light duration of 12 h. All experimental procedures involving rats were approved by the Institutional Animal Care and Use Committee of The Fourth Affiliated Hospital of Jiangsu University. Five-week-old SD female rats were intraperitoneally injected with 50 mg/kg CTX (Sigma-Aldrich, St. Louis, MO, USA) on the first day and then with 8 mg/kg/d CTX consecutively for 14 days as described in the literature. We randomly divided the rats into five equal groups with ten rats in each group. The WT group consisted of normal control rats that received no treatment. In the POF group, the rats were administered CTX. In the PBS group, POF rats were injected intraperitoneally with 100 μL of PBS every other day for 2 weeks. In the BMSC group, POF rats were injected intraperitoneally with 1 × 10^6^ BMSCs in a 100 μL volume of PBS every other day for 2 weeks.

**1.2 The plasmid constructs (source, species, isoforms, tag N- or C-terminal) as well as for the shRNAs**

Plasmid construction and small interfering RNA synthesis. SIAH1(NM_001006610), Human, CMV-MCS-3FLAG-EGFP-SV40-Neomycin,SIAH1(63929-1)-p1:5’ACGGGCCCTCTAGACTCGAGCGCCACCATGACGGGAAAGGCTACTCC3’,SIAH1(63929-1)-p2:5’AGTCACTTAAGCTTGGTACCGAACACATGGAAATAGTTACATTGATG-3’.SIAH1(NM_001006610), Human CMV-MCS-3FLAG-SV40-Neomycin. The SIAH1-RNAi (6691-1),hU6-MCS-CMV-GFP-SV40-Neomycin,human,SIAH1-RNAi(6691-1)-a:5’-GATCCCGATAGGAACACGCAAGCAA-CTCGAG-TTGCTTGCGTGTTCCTATC-TTTTTGGAT-3’,SIAH1-RNAi(6691-1)-b:5’-AGCTATCCAAAAA-GATAGGAACACGCAAGCAA-CTCGAG-TTGCTTGCGTGTTCCTATC-GG-3’ were synthesized by Genechem, Shanghai, China). TRF2, Human, PRK5-FLAG-TRF2-Amp, was presented by Dr. Li Lan from the Cancer Center Laboratory of Massachusetts General Hospital.

**1.3 Western blotting**

Sodium dodecyl sulfonate (SDS) lysis buffer (#R0020, Solarbio) with Protease Inhibitor Cocktail (#2958490, Millipore,USA) was added into ovarian tissues and cells after three times washed with ice-cold PBS. About 60g of protein in each lane was separated by SDS polyacrylamide gel, and then transferred to a polyvinylidene fluoride membrane. Membrane was sealed in 5% skimmed milk for 2 hours at room temperature incubated with SIAH1(#ab2237, Abcam), TRF2 (#ab2357, Abcam), P53(#WL02504, Wanleibio), Tubulin (#2125, CST) and ubiquitin (sc-8017, Santa Cruze Biotechnology) primary antibodies following horseradish peroxidase-conjugated secondary antibodies (#14708, CST; #ab97110; Abcam; #14709, CST). An ECL chemiluminescence kit (#E412-01, Vazyme) was used to detect immunoreactive protein bands by ChemiDoc MP Imaging System.

**1.4 Real-time quantitative RT–PCR.**

Total RNA was extracted with TRIzol reagent (#262304, Invitrogen) according to the manufacturer’s protocol. Complementary DNA (cDNA) was synthesized using the FastQuant RT (with gDNase) (#KR106, Tiangen, Shanghai, China). Real-time polymerase chain reaction (qRT-PCR) analyses were performed using a mRNA qPCR Detection Kit (#B532954, Sangon Biotech). The mRNA expression levels were analyzed by the 2−ΔΔCT method and normalized to those of β-actin, individually. The primer sequences were 5’-GACTCCTCGGTCTATTCATGAG-3’ and 5’-CCATTTTCTGCAAAAAGCTGTG-3’ for SIAH1. Supplementary Material should be uploaded separately on submission. Please include any supplementary data, figures and/or tables. All supplementary files are deposited to FigShare for permanent storage and receive a DOI.

# Supplementary Figures


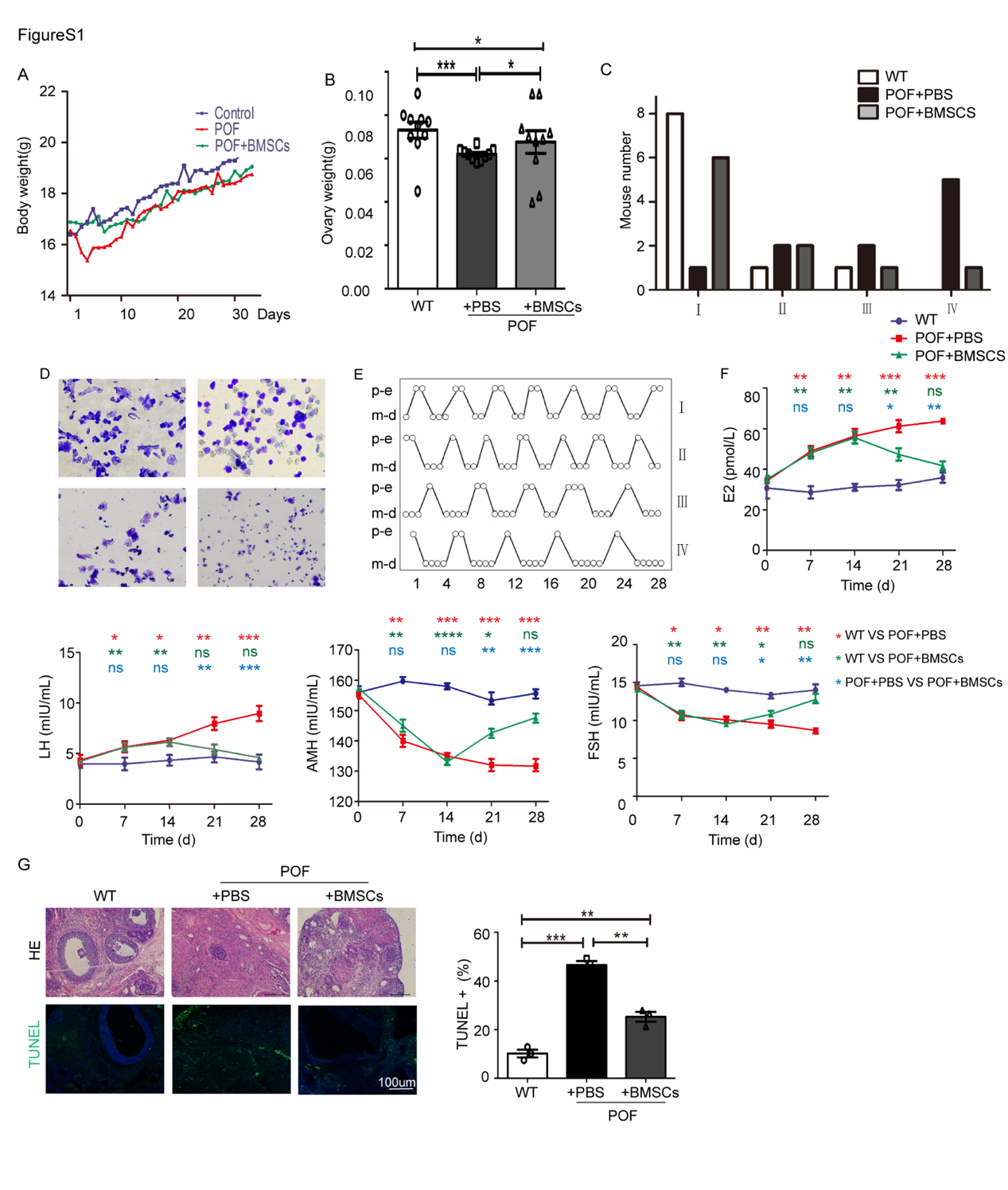


**Supplementary Figure 1.** **A successful in vivo model of POF in rats. (A)** Body weight (g) and **(B)** ovary weight (g) of the three groups before and after CTX treatment compared at the same time. **(C)**Total numbers of rats from each group categorized into the various estrous patterns (I–IV). **(D)**Normal estrous cycles: 1, proestrus; 2, estrus; 3, metestrus; 4, diestrus. **(E)** Four patterns of estrous cycles were graded based on the severity of abnormality (I–IV): I, normal; II, regular cycles with shortened estrus; III, irregular cycles with prolonged diestrus and normal or prolonged estrus; IV, no cyclicity. The y axis represents the cycle day in proestrus or estrus (p-e) and metestrus or diestrus (m-d). Each circle (○) represents one rat. The illustration represents only one rat and is meant to show the change in estrus cycle. **(F)**Trends for the average levels of E2, LH, AMH. and FSH. Comparison of the AMH, E2, FSH, and LH levels between groups at different times. **(G)** Hematoxylin and eosin assay of GC apoptosis. The green stain indicates tunnel-positive GCs. The blue stain indicates the nucleus. staining. TUNEL assay of GC apoptosis. The green stain indicates tunnel-positive GCs. The blue stain indicates the nucleus.


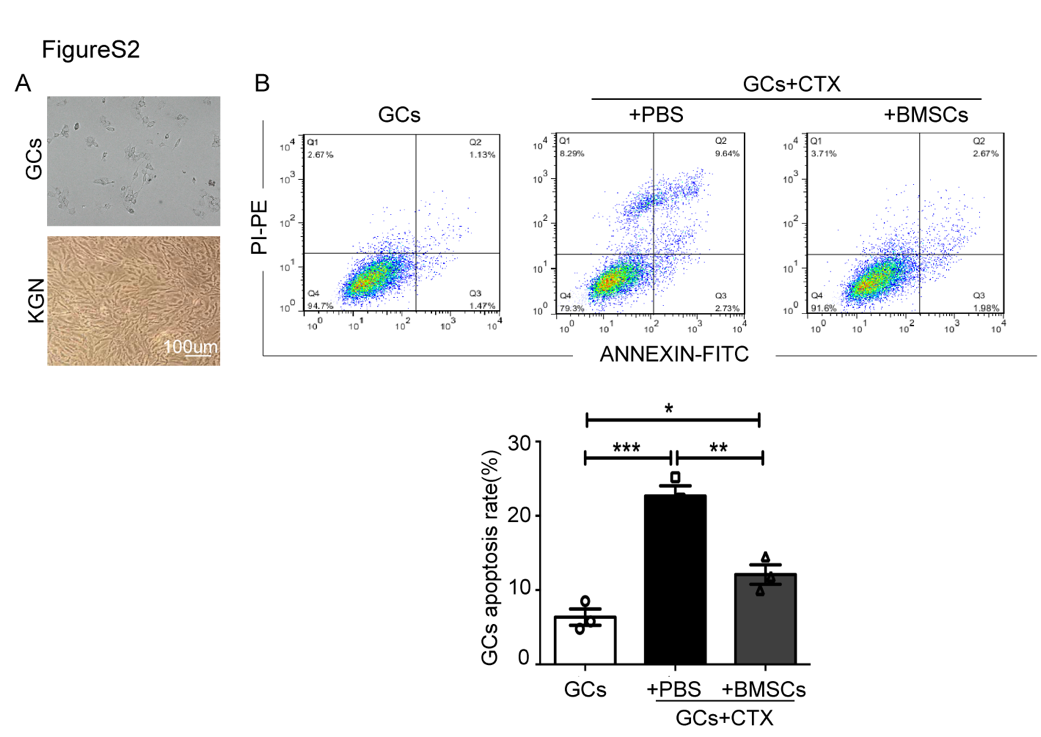


**Supplementary Figure 2.** **A successful establishment of the POF model in GCs. (A)** Morphology of GCs and KGN cells. **(B)** The apoptosis rates of GCs as determined by flow cytometry.


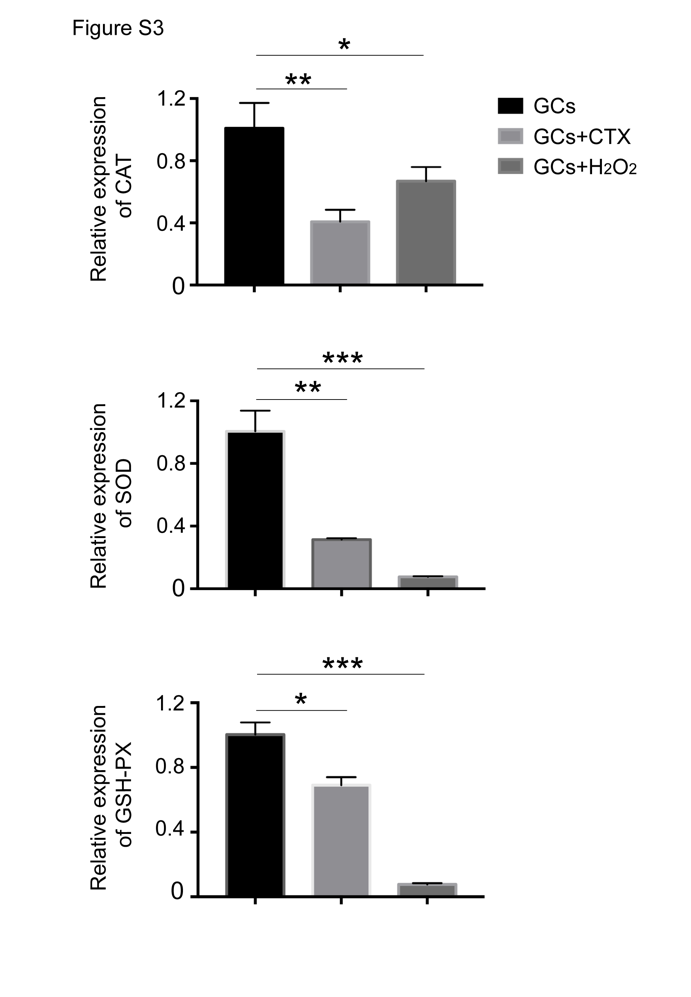


**Supplementary Figure 3.** Oxidative response to H2O2 stimulation was determined by PCR, the histograms show the relative expression of CAT, GSH-Px and SOD2 in GCs. Histograms of statistical difference between groups are shown.


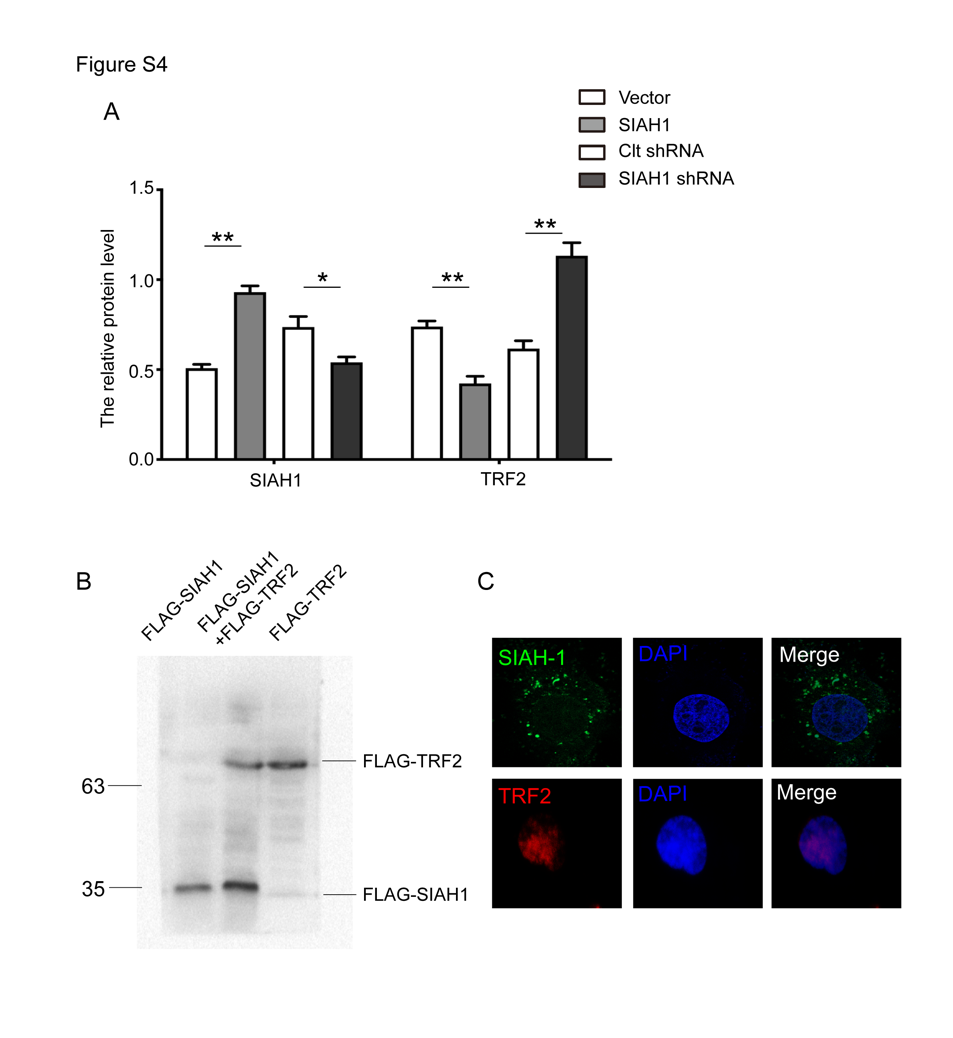


**Supplementary Figure 4. (A)** Statistics of SIAH1 and TRF2 related expression in Fig.4A . **(B)** WB image verification of FLAG-SIAH1 and FLAG-TRF2 vectors. **(C)**The GFP-SIAH1 or FLAG-TRF2 expression plasmids were transfected separately into KGN cells. GFP-SIAH1 was detected using a fluorescence microscope with an excitation wavelength of 488 nm. FLAG-TRF2 was detected with an excitation wavelength of 556 nm. The cell nuclei were stained with DAPI.
